# Use of closed suction drain after primary total knee arthroplasty – an overrated practice

**DOI:** 10.1051/sicotj/2016034

**Published:** 2016-11-18

**Authors:** Gaurav M. Sharma, Gauresh Palekar, Dilip D. Tanna

**Affiliations:** 1 Lotus Hospital Mama Parmanand Marg 400004 Mumbai Maharashtra India

**Keywords:** Drainage, Arthroplasty, Replacement knee, Length of stay

## Abstract

*Purpose*: The age-old practice of closed suction drain following orthopedic procedures has been challenged since past few decades. Our aim was to assess the effectiveness of closed suction drain after total knee arthroplasty.

*Materials and methods*: One hundred twenty patients (135 knees) with primary Total Knee Arthroplasty were divided into a study group (no drain) and a control group (drain used). Inclusion criteria were grade 3 and grade 4 osteoarthritis of the knee. Revision cases and rheumatoid arthritis were excluded. Parameters assessed were pain, pre and post-op Hb, dressing change, early infection, ecchymosis and duration of stay. Results were calculated using Western Ontario and McMaster Universities Osteoarthritis Index and Oxford Knee scoring systems at two weeks, six months and one year.

*Results*: Mean age was 72.03 ± 6.68 in study group and 71.38 ± 7.02 in control group. Pre and post op Hb was 12.1678 ± 1.3220 (study group), 12.1803 ± 1.2717 (control group) and 9.8373 ± 1.5703 (study group), 9.7918 ± 1.4163 (control group). There was one case of early infection in both groups which was controlled by oral antibiotics. Change of dressing and ecchymosis were more in the study group. Duration of hospital stay was more in the control group *p* < 0.0006 (statistically significant).

*Conclusion*: There is no added advantage of closed suction drain over no drain usage and this practice can safely be brought to a halt.

## Introduction

Closed suction drain is being used widely in different surgical specialties, with orthopedics not being an exception. The use of suction drain has been practised routinely, ever since the era of Hippocrates. Draining the wounds has been a matter of controversy and discussion over the past few decades with more advanced sterility techniques and decreased infection rate. Many surgeons blindly follow the practice as per their early training. The rationale behind the use of suction drain is thought to be the prevention of hematoma formation, which is one of the factors responsible for colonization by bacteria, thus leading to superficial as well as deep-seated infection [1]. On the contrary, many studies have proved that there is retrograde migration of the bacteria from the drain lumen into the wound causing deep-seated infection [2, 3]. Drains help in reducing the post-operative ecchymosis and the need for reinforcement of the dressings associated with no drain usage. On the other hand, many studies have shown more blood loss and the need for blood transfusion post-operatively among the drain users [3–5].

## Materials and methods

One hundred and twenty consecutive patients (135 knees) who underwent primary cemented Total Knee Arthroplasty between May 2014 and May 2015 were included in the study. Inclusion criteria were patients with grade 3 and grade 4 osteoarthritis of the knee. Patients with revision arthroplasties, patients on prior anticoagulant therapy, and patients with rheumatoid arthritis were excluded from the study. Fifty-nine patients (nine bilateral) were included in the study group (no drain used) and 61 patients (six bilateral) were in the control group (drain used). The patients were followed up post-operatively at the second week, six months, and at one year. One patient in the control group missed the six-month follow-up but was still included in the study as he turned up for the one-year follow-up.

All the patients were selected by simple randomization by using a closed envelope technique. The patients were asked to open the envelope just prior to the surgery. Well-written informed consent was taken from all the patients enrolled in the study. Prior Ethics Committee’s approval was obtained for the study. Spinal, combined with epidural anesthesia was used in the majority of cases. The standard surgical steps were followed. All the patients were given three doses of second-generation cephalosporin (one within 30 min before the procedure and two doses at 12-hour interval post-operatively). Three doses of 1 g intravenous tranexamic acid (one pre-operatively and two post-operatively at 12-hour interval) were given to all the patients. Anterior midline incision and medial parapatellar approach were used for all the cases. Pneumatic tourniquet was used in all the cases and was deflated prior to closure to catch the bleeders. Local infiltration with 0.5% sensorcaine (bupivacaine), 2 mL ketorolac (nonsteroidal anti-inflammatory drug, NSAID), and 80 mg of tobramycin (aminoglycoside) diluted in 30 mL of normal saline was infiltrated locally in each knee just before cementing. NexGen^®^ LPS-Flex (Zimmer, Warsaw, USA) was used in all the cases. Patella was not replaced in any of the cases. Bone cement Palacos^®^ LV (Zimmer, Warsaw, USA) was used in all the cases. The closed suction drain used in the control group was Number 10/12 Romo Vac^®^ (Romsons, India). Thick compression dressing was done in all the patients post-operatively. Similar pain management protocols were followed in both the groups. Drain removal in all the patients was done after 24 h, except in one case of Bilateral Total Knee Arthroplasty where the drain was removed on the third post-operative day due to persistently large volume of collection.

The primary outcome measures assessed were pain, pre- and post-operative hemoglobin, dressing change within 24 h, range of motion, early infection, discharge from the wound/drain site, ecchymosis around the wound, and the duration of hospital stay.

The final results were assessed using the Western Ontario and McMaster Universities Osteoarthritis Index (WOMAC) and The Oxford Knee scoring (OKS) systems.

## Statistics

Fisher’s Exact test was used to assess the parameters such as discharge from the wound, ecchymosis, early infection, and decrease in the range of movements. Two sample independent *t*-test was used to assess the pre- and post-operative hemoglobin and the average duration of hospital stay. The results were expressed as mean with standard deviation and *p* < 0.05 was considered to be statistically significant. Analysis was done using the Epi-info software (Version 3.4.3) and Microsoft Excel 2013 (Microsoft Office v15.0).

## Results

The mean age of the patients was 72.03 ± 6.68 in the study group and 71.38 ± 7.02 in the control group. Nine patients in the study group and six patients in the control group underwent simultaneous Bilateral Total Knee Replacement.

The mean pre- and post-operative hemoglobin, dressing change with 24 h, ecchymosis, early infection, range of movements, and the average duration of the hospital stay are summarized in Table 1.

**Table 1.** Different variables like hemoglobin (Hb), dressing change, early infection, ecchymosis, decreased range of movements, and duration of hospital stay.

Variable	Study group (*n* = 59)	Control group (*n* = 61)	Test of significance used	*p* value
Pre-operative hemoglobin	12.1678 ± 1.3220	12.1803 ± 1.2717	Two sample independent *t*-test	0.9563
Post-operative hemoglobin	9.8373 ± 1.5703	9.7918 ± 1.4163	Two sample independent *t*-test	0.8681
Dressing change within 24 h	7 (11.8)	3 (4.9)	Fisher’s exact	0.2043
Early infection	1 (1.69)	1 (1.63)	Fisher’s exact	1.0000
Ecchymosis	3 (5.08)	2 (3.2)	Fisher’s exact	0.6769
Decreased range of movements	2 (3.3)	6 (9.8)	Fisher’s exact	0.2727
Duration of hospital stay	5.12 ± 1.65	6.21 ± 1.74	Two sample independent *t*-test	0.0006*

* Statistically significant.

There was a statistically significant difference in the duration of hospital stay (*p* < 0.0006) with the control group requiring a longer stay in the hospital.

The WOMAC and the OKS systems scores were assessed pre-operatively, at two weeks, six months, and at the end of one year, the values of which are summarized in Tables 2 and 3, respectively. There was no statistically significant difference found in both the scores.

**Table 2.** WOMAC index.

Variables	Drain (*n* = 61)	No drain (*n* = 59)	*t*-value	*p* value
Pre-Op	12.3623 ± 4.0016	13.0017 ± 4.3021	0.8418	0.4016
Two weeks	30.1721 ± 5.1560	30.1814 ± 5.1047	0.0096	0.9924
Six months	52.1593 ± 7.3826	52.8770 ± 9.9231	0.4485	0.6547
One year	83.6443 ± 8.7956	83.8966 ± 8.4728	0.1599	0.8733

**Table 3.** Oxford knee scoring (OKS).

Variables	Drain control (*n* = 61)	No drain study (*n* = 59)	*t*-value	*p* value
Pre-Op	10.796 ± 2.8814	10.389 ± 3.0739	0.7478	0.4561
Two weeks	24.1803 ± 3.7617	24.1356 ± 3.6410	0.0666	0.947
Six months	34.48 ± 4.1066	33.9661 ± 3.8639	0.7064	0.4814
One year	43.2787 ± 3.4647	43.2712 ± 3.5807	0.0109	0.9913

## Discussion

The practice of closed suction drain is constantly being challenged by many surgeons worldwide with a decline in its use. Hematoma formation and thus the fear of subsequent infection has been the prime reason for the use of closed suction drain. It is the age-old belief that draining the wound prevents the increased collection of blood in different compartments of the wound post-operatively which is the cause of hematoma formation. Hematoma being a good culture medium for the bacteria with low level of opsonic proteins hinders the normal phagocytic activities and also delays the normal wound healing process [1, 5]. Theoretically, drain usage does not allow the tamponade effect to occur which is an important step in filling the dead space in an operated wound. Various methods have been used in order to determine the level of post-operative hematoma formation namely weighing the post-operative dressings [6, 7], using ultrasound of the wound [8] and single-photon emission computed tomography [9]. However none of these methods has shown that there is a major role of the drain in the prevention of haematoma formation. On the contrary, there is always some amount of hematoma which is left unevacuated in the wound irrespective of drain usage. Thus there seems to be no logical reason to use the drain in terms of hematoma prevention.

Infection is one of the major complications following Total Knee Arthroplasty and this requires immediate attention. There was one patient in each group in our study with early superficial infection, and both resolved completely with oral antibiotics. A recent meta-analysis showed 0.5% incidence of infection in the drain group and 1.2% incidence of infection in the non-drained group [10] which was similar to a randomized trial showing the rate of superficial infection in 2.9% and 4.8% in drained and non-drained group, respectively [3]. However, we did not find any statistically significant difference in terms of superficial wound infection between our groups. There was no case of deep wound infection in our study.

**Figure 1.**
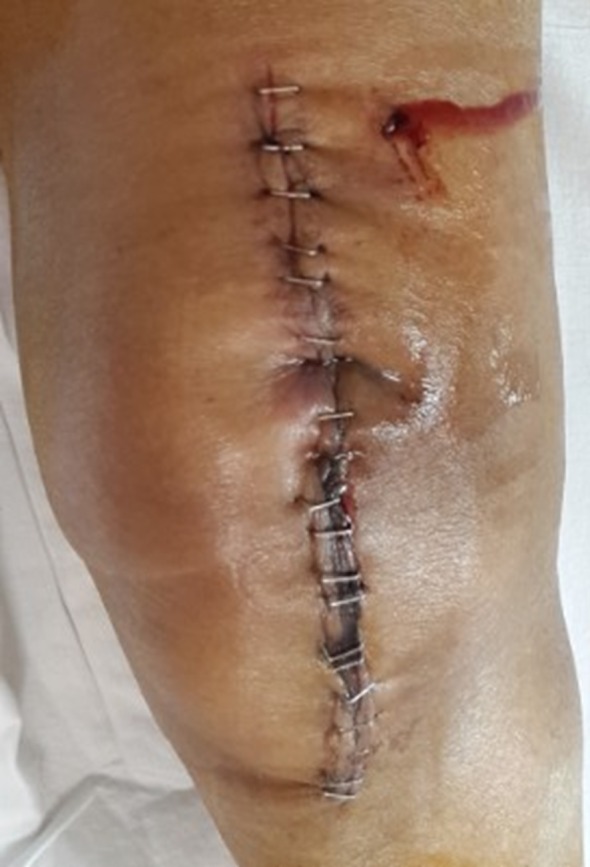
Discharge from the drain site.

Persistent discharge from the wound site [6] and ecchymosis [6, 11] have been found to be statistically significant in a few studies where drain were not used. Although more patients in the study group required a change of dressing in the first 24 h and had ecchymosis when compared to the control group, the final results were not statistically significant in our study. There was one patient (1.69%) in the study group, in whom the serous discharge from the wound lasted up to the 7th post-operative day. The discharge from the wound was typically bloody with fluid in the initial few days owing to the presence of hematoma, which later turned serous after 3–4 days. Similarly, there were two patients (3.2%) in the control group with serous discharge from the drain insertion site lasting till the 8th and 10th post-operative days, respectively (Figure 1). Discharge from the wound or the drain insertion site further adds to the total cost.

Blood loss is another major and important concern following total knee arthroplasty, the majority of the loss occurring in the first few hours post-operatively [12]. The reason behind this is that there is increased reactive blood flow in the wound, once the tourniquet is deflated. There is an associated risk of blood loss leading to blood transfusion especially in the patients where a drain has been used. There was one patient (1.6%) with Bilateral Total Knee Arthroplasty in the control group who required blood transfusion post-operatively but this may be related to low pre-operative hemoglobin level. There was no statistically significant difference in both the groups in terms of change in the hemoglobin levels both pre- and post-operatively. Various methods have been used in the past and present to control the blood loss post-operatively in patients where a drain is used, one of which is temporary clamping of the drain. This method not only allows hematoma formation at a later stage, but also allows the tamponade effect to occur which is required to fill up the dead space. The duration of drain clamping and time for the drain removal have also been a matter of debate over the past few years with majority of the studies suggesting that initial clamping for 4–6 h has a better effect [13]. Post-operatively, the drain should not be kept beyond 48 h [14] as it further increases the risk of retrograde migration of the bacteria. The practice of clamping the drain for a long period is similar to not draining the wound. In the present study, no clamping was done in the control group patients and all the drains were removed after 24 h (except one which was removed at third post-operative day as mentioned earlier).

It has been our observation that a drain interferes with post-operative physiotherapy and the nursing care thus delaying the mobilization which further adds to the duration of hospital stay which was significant in our study (*p* < 0.0006). Our finding was similar to those of other studies [5, 15]. The presence of drain interferes with the post-operative knee range of movements to some extent which itself contributes to an increase in the duration of hospital stay.

The outcome measures were calculated with the help of WOMAC and OKS systems which have a very good predictive value, are simple to use. They are easily available which helps to measure the outcomes of the total knee replacement (TKR) in regard to other non-surgical interventions [16]. The results of both the scores were not statistically significant in our study.

The limitations of our study were: the small sample size, short duration of follow-up, and the presence of few confounding variables like similar pain control protocols in both the groups and use of intravenous tranexamic acid to control the bleeding both intra- and post-operatively.

## Conclusion

We are of the opinion that the age-old practice of draining the wound has no effect on the post-operative control of pain, infection, and blood loss. On the other hand, it raises the risk of infection if used for a longer time. Although no drain usage is associated with more dressings post-operatively with a greater area of ecchymosis, the benefits of drain usage are still very limited. Thus, there is no added advantage of closed suction drain over no drain usage in primary Total Knee Arthroplasty and this practice can safely be brought to a halt.

## Conflicts of interest

The authors declare no conflict of interest in relation with this paper.
